# Interdisciplinary team approach for complicated type B aortic dissection with concomitant hematothorax by endovascular stent grafting and left side mini thoracotomy: a case report

**DOI:** 10.1186/1749-8090-7-111

**Published:** 2012-10-12

**Authors:** Marcel Vollroth, Joerg Seeburger, Philipp Kiefer, Michael Hoebartner, Yvonne Bausback, Jens Garbade, Lukas Lehmkuhl, Friedrich Wilhelm Mohr

**Affiliations:** 1 Department of Cardiac Surgery, Heartcenter Leipzig University, Struempellstrasse 39, Leipzig, 04289, Germany; 2 Center of Vascular Medicine, Angiology and Vascular Surgery, Park Hospital Leipzig, Leipzig, Germany; 3 Department of Diagnostic and Interventional Radiology, Heartcentre Leipzig University, Leipzig, Germany

**Keywords:** Aortic dissection type B, Endovascular stent grafting, Interventional treatment

## Abstract

Due to high mortality rates in surgical treatment, total endovascular stent grafting has become a promising therapeutic option in patients with acute aortic dissection type B. In our case, a 76- year- old patient with acute ruptured aortic dissection type B and hematothorax achieved concomitant total endovascular stent grafting and left side mini thoracotomy. With moderate neurologic impairment he was discharged from hospital after 20 days.

This case shows that early mortality of live threatening acute aortic dissection type B with hemorrhagic pleural effusion may be reduced by total endovascular stent grafting and concomitant mini thoracotomy.

## Background

Currently, there are still different ideas about the management of acute type B aortic dissections. Generally, medical treatment is preferred when there are no life-threatening complications [1]. In contrast surgical intervention for acute type B aortic dissection has been reserved for complications such as aneurismal rupture, end organ malperfusion and failure of conservative management. In such special cases mortality ranges from 25-50% [2]. Endovascular aneurysm repair (EVAR) which was first applied to abdominal aortic aneurysms in the early 1990s and to descending aorta aneurysms later on, has currently achieve the treatment of choice for acute type B dissection [2]. Several studies demonstrated technical feasibility of endovascular approaches even to tackle the difficult clinical scenario of type B dissection [3-5]. However, we suggest that patients may benefit from surgical stand by in the event of acute blood loss or dramatic aortic rupture.

In this report, we present a case of Thoracic endovascular aortic repair (TEVAR) and concomitant left side mini thoracotomy for the treatment of acute type B aortic dissection in a patient with acute hemorrhagic shock due to severe hematothorax.

## Case presentation

**Figure 1:**
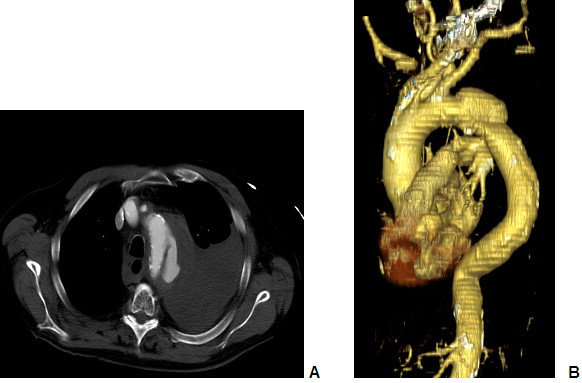
A+B: Computed tomography scans obtained at admission ruptured dissection type B and extensive left-sided hematothorax.

A 76-year-old male patient diagnosed ambulant with acute ruptured aortic type B dissection was referred to our ICU. He presented with severe tearing pain that migrated from his back and then to his abdomen. He had a history of hypertension, diabetes and hyperlipidemia. Physical examination upon arrival revealed blood pressure of 82/55 mmHg. The patient complained distress and vomiting. Three dimensional reconstruction of the CT angiography showed a dissecting and acute ruptured aneurysm from 1 cm beyond the left subclavian artery with a diameter reaching 4 cm at the largest part. There was also distinctive left side hematothorax (Figure 1). Fortunately, all abdominal arteries were feeded by the true lumen with no signs of abdominal malperfusion. Laboratory examination showed highly increased lactate level while haemoglobin was decreased. Based on the severe hemorrhagic shock, the patient was immediately transferred to our hybrid OR. Under general anaesthesia the patient needed high dose inotropic support and showed poor oxygenation despite high pressure ventilation. Our interventional team, consisting of two angiologists and one heart surgeon, decided to release the left pleura due to a 4cm left side mini thoracothomy in the 4^th^ intercostal space and overall delivered three litre fluid hematoma.

Simultaneously the left femoral artery was surgically accessed and a transversal arteriotomy was performed. A 5F sheath was inserted percutaneously through the right brachial artery, through which a 5F pigtail catheter was directed for angiographic monitoring of the endovascular graft position. After administering 5,000 units of heparin we inserted the delivery system through the arteriotomy in the left common femoral artery. Valiant Captiva (34-34-200 mm) stent prosthesis was positioned and expanded in order to exclude aneurysm completely. At the end of the procedure, angiography was used to demonstrate the correct location of the stent and regular perfusion of the aorta and its branches.

With moderate inotropic support, the patient was administrated to our ICU. Furthermore the patient received a cerebrospinal fluid drainage system immediately after arriving on ICU. It was used for spinal cord protection within the next 3 days.

**Figure 2:**
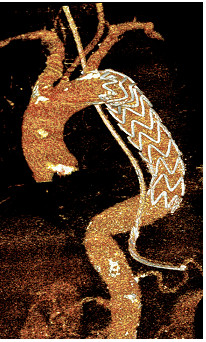
CT scan reconstruction shows regular closure of the entry site by the stent graft.

The post interventional course was complicated by renal failure and neurologic morbidity necessitating tracheotomy. Computed tomographic scan showed mild ischemia in the area of the left middle cerebral artery. We suppose that the meanwhile low flow during stenting procedure was causal for renal und neurologic impairment. Fortunately all neurologic deficits resolved moderate within the next days. On post interventional day 20 the patient was transferred to a neurologic rehabilitation. He has been followed up regularly and was free of neurologic symptoms after six months. Three dimensional reconstructions of the control- CT scan showed regular stent expansion without any endoleak (Figure 2).

### Commentary

The suitable treatment strategy for acute descending aortic dissection has long been a matter of debate and continues to be a challenge [1]. High mortality rates in surgical treatment (25-50%) of complicated acute type B dissections, directed surgeons to search for other treatment modalities. Implementation of endovascular techniques has provided new therapeutic options [5]. Initial series and subsequent multicenter trials demonstrated technical feasibility and a low rate of complications even in high-risk patients with acute type B dissection.

However, treatment of acute aortic dissections by endovascular grafting itself carries some risks. Leakage can occur in approximately 25% of patients. Rarely, the stent graft may not plug the aortic wall adequate and may dislocate. In 8% of patients embolic material may originate from an atherosclerotic basis and corrupt the blood flow of the spinal cord, leading to paraplegia. There is furthermore the risk for abdominal malperfusion. In this situation fenestration of the dissection membrane is recommended in several publications.

The most serious circumstance is rupture of the dissected aorta. In most of these cases the blood loss is very high and occurs into the left pleura. Without sufficient treatment strategies in specialized centres it leads directly to subsequent hemorrhagic shock and highly increased morbidity and mortality. This case illustrates that optimal treatment strategies are necessary to avoid serious complications. In our case, hemorrhagic and pulmonary shock due to ruptured aneurysm requires simultaneous thoracotomy and endovascular stent implantation. While opening the left pleura and release hemorrhagic effusion, stent grafting was performed during sufficient circulation and mechanical ventilation.

## Conclusion

Complicated acute type B dissection remains a clinical challenge. Patients with complicated type B dissection and signs of clinical instability at presentation have a high risk of in-hospital mortality. The choice of endovascular stent-graft placement in combination with surgical stand by may offer a strategy to improve in-hospital prognosis.

## Consent

Written informed consent was obtained from the patient for publication of this report and any accompanying images.

## Competing interests

The authors declare that they have no competing interests.

## Authors’ contributions

JG and YB performed the operations. MV wrote the manuscript. JS helped revise the manuscript. All authors read and approved the final manuscript.
